# Antimicrobial Activity of Zabofloxacin against Clinically Isolated *Streptococcus pneumoniae*

**DOI:** 10.3390/molecules21111562

**Published:** 2016-11-17

**Authors:** Hee-Soo Park, Sang-Hun Oh, Hye-Shin Kim, Dong-Rack Choi, Jin-Hwan Kwak

**Affiliations:** 1 School of Food Science and Biotechnology, Kyungpook National University, Daegu 41566, Korea; phsoo97@knu.ac.kr; 2 School of Life Science, Handong Global University, 558 Handong-ro, Buk-gu, Pohang 37554, Korea; osh8755@naver.com (S.-H.O.); momobile217@gmail.com (H.S.K.); 3 Dong Wha Pharm., Ind. Co. Ltd., Anyang 31041, Korea; dongrack.choi@dong-wha.co.kr

**Keywords:** zabofloxacin, minimum inhibitory concentration, *Streptococcus pneumoniae*, in vivo

## Abstract

Zabofloxacin is a novel fluoroquinolone agent that has potent activity against gram-positive pathogens. In this study, we confirmed that zabofloxacin showed the most potent in vitro and in vivo activities against drug-resistant *Streptococcus pneumoniae*. Among the fluoroquinolone compounds, zabofloxacin showed the most potent in vitro activity against clinical isolates of penicillin-sensitive *S. pneumoniae* (minimum inhibitory concentration, MIC_90_: 0.03 mg/L) and penicillin-resistant *S. pneumoniae* (MIC_90_: 0.03 mg/L). Against quinolone-resistant *S. pneumoniae,* zabofloxacin (MIC_90_: 1 mg/L) was more active than ciprofloxacin, sparfloxacin, and moxifloxacin; however, its activity was the same as that of gemifloxacin. The in vivo activity of zabofloxacin was most potent among the quinolone compounds tested against the systemic infection and respiratory tract infection models in mice.

## 1. Introduction

*Streptococcus pneumoniae* is one of the most important pathogenic bacteria known to cause community-acquired pneumonia, acute otitis media, and meningitis, with high morbidity and mortality rates [1]. Various antibiotics including beta-lactams and macrolide agents are used to treat bacterial pneumonia; however, the emergence of antibiotic-resistant bacteria has been rapid worldwide [2]. In the United States, 30% of *S. pneumoniae* strains are resistant to one or more antibiotics including penicillin, other beta-lactams, and macrolide agents [3,4]. Therefore, fluoroquinolones and ketolides have been considered for the treatment of multi-drug resistant pneumococci [5].

Zabofloxacin (DW-224a) is a novel fluoroquinolone antibiotic with potent antibacterial activity against gram-positive cocci [6,7]. Previous studies demonstrated that zabofloxacin had effective in vitro activity against drug-resistant *S. pneumoniae* isolates that caused non-invasive and invasive disease [6,8,9]. In this study, we further examined the in vitro activities of zabofloxacin against quinolone-susceptible (QSSP) and quinolone-resistant *S. pneumoniae* (QRSP) strains when compared to other antimicrobial agents. Moreover, we found that zabofloxacin was the most potent antibacterial agent against penicillin-resistant *S. pneumoniae* (PRSP) in the murine systemic infection model used in this study.

## 2. Results

The minimum inhibitory concentrations (MICs) of compounds tested against QSSP are presented in Table 1. The MIC_90_ of zabofloxacin was 0.03 mg/L (MIC_90_ is the concentration at which 90% of the strain growth is inhibited), which is the lowest among the compounds tested, followed by gemifloxacin (MIC_90_: 0.03 mg/L), moxifloxacin (MIC_90_: 0.25 mg/L), sparfloxacin (MIC_90_: 0.5 mg/L), and ciprofloxacin (MIC_90_: 2 mg/L). QSSP strains were also found to be susceptible to linezolid (MIC_90_: 1 mg/L) and synercid (MIC_90_: 1 mg/L). These results indicated that zabofloxacin was most potent among the agents tested against QSSP strains.

Against 22 QRSP strains (ciprofloxacin MICs ≥8.0 mg/L) that contain a mutation in the quinolone resistance-determining region (QRDR), zabofloxacin (MIC range: 0.06–2 mg/L; MIC_90_: 1.0 mg/L) was more active than ciprofloxacin (MIC range: 4–64 mg/L; MIC_90_: 64.0 mg/L) and moxifloxacin (MIC range: 4–8 mg/L; MIC_90_: 8.0 mg/L), and as active as gemifloxacin (MIC range: 0.06–2 mg/L; MIC_90_: 1.0 mg/L) (Table 2). All 22 strains contain 2 or more mutations in the QRDR of *gyrA*, *parC*, and/or *parE*, but not *gyrB* (Table 3). In the presence of reserpine, an efflux pump inhibitor [10], 3 out of 22 strains exhibited MIC lower than that of ciprofloxacin and 1 strain exhibited MIC lower than that of gemifloxacin. However, all isolates showed the same MIC for zabofloxacin and moxifloxacin in the presence of an efflux inhibitor (Table 3). These results demonstrated that zabofloxacin is effective against mutation in the QRSP of target genes and a poor substrate for efflux pumps.

**Figure 1:**
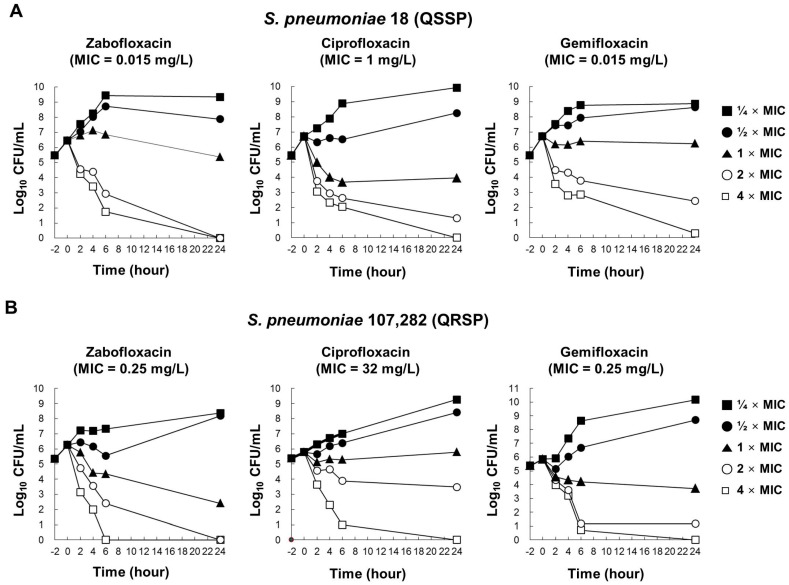
Time-kill curves of zabofloxacin, ciprofloxacin, and gemifloxacin against *Streptococcus pneumoniae*. (**A**) *S. pneumoniae* 18 (quinolone-susceptible *S. pneumoniae*, QSSP) exposed to zabofloxacin, ciprofloxacin, and gemifloxacin; (**B**) *S. pneumoniae* 107,282 (quinolone-resistant *S. pneumoniae*, QRSP) exposed to zabofloxacin, ciprofloxacin, and gemifloxacin.

To examine the bactericidal activity of zabofloxacin against *S. pneumoniae*, time-kill analysis was carried out. As shown in Figure 1, zabofloxacin and gemifloxacin showed rapid bactericidal activity at 2 and 4 times the MIC against QSSP and QRSP. Ciprofloxacin showed bactericidal activity at 4 times MIC against QRSP only, but this concentration of ciprofloxacin (128 mg/L) could not be used for the treatment of *S. pneumoniae*. The regrowth of tested strains was completely inhibited by zabofloxacin.

**Figure 2:**
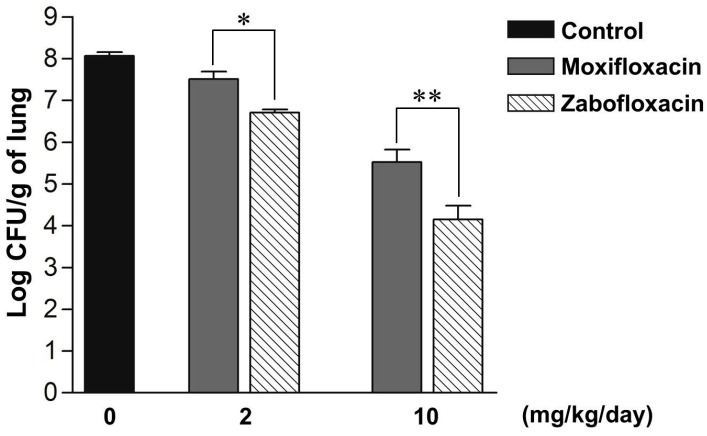
Therapeutic efficacies of zabofloxacin and moxifloxacin in a respiratory tract infection model caused by *S. pneumoniae* 1065 (PRSP). The number of viable *S. pneumoniae* in the lungs was examined after infection with PRSP and administration the test drugs. Each bar represents the mean ± SEM (standard error of the mean) (*n* = 4). Zabofloxacin significantly reduced the number of bacteria compared with moxifloxacin (* *p* < 0.05; ** *p* < 0.01).

Previous studies reported that the zabofloxacin showed the most potent activity against penicillin-susceptible *S. pneumoniae* (PSSP) [6,7]. In this study, we examined in vivo efficacies of zabofloxacin against systemic infections and compared the results with those of ciprofloxacin, moxifloxacin, and gemifloxacin against PRSP 1065 strain. Zabofloxacin was the most potent quinolone (MIC: 0.015 mg/L; ED_50_ (median effective dose): 0.42 mg/kg), followed by ciprofloxacin (MIC: 4 mg/L; ED_50_: 31.45 mg/kg), moxifloxacin (MIC: 0.25 mg/L; ED_50_: 18.00 mg/kg), and gemifloxacin (MIC: 0.03 mg/L; ED_50_: 2.09 mg/kg) (Table 4). These results agreed well with the in vitro MIC values. They indicated that zabofloxacin exhibits the most potent protective effects against systemic infections caused by penicillin-susceptible *S. pneumoniae* and PRSP. We then examined the in vivo effect using the pneumococcal pneumoniae model. As shown Figure 2, zabofloxacin significantly reduced bacterial counts in the lung compared with moxifloxacin.

## 3. Discussion

Zabofloxacin is a novel fluoronaphthyridone quinolone with a 7-pyrrolidone substituent that showed excellent in vitro activities against both gram-positive and gram-negative strains [6,7]. Importantly, zabofloxacin has been shown to have excellent in vivo activity against gram-positive pathogens including *Steptococcus aureus (S. aureus)*, *Streptococcus pyogenes (S. pyrigenes),* and *S. pneumonia* [6,7]. Zabofloxacin also was very active against pathogenic bacteria that cause community-acquired respiratory tract infections, including *Haemophilus influenzae* and *Moraxella catarrhalis* [6,7]. In addition, zabofloxacin could be considered as an alternative candidate for treatment of quinolone-susceptible and quinolone-resistant gonorrhea [11]. Therefore, zabofloxacin is considered a potent antibacterial candidate for clinical trials and has been approved in South Korea for specific treatments [8,12].

Several studies have suggested that zabofloxacin has potent activity against non-invasive and invasive *S. pneumoniae* [6,7,8] and has bactericidal activity against several *S. pneumoniae* strains [9,13]. This compound targets DNA gyrase and topoisomerase IV, and mutations in both proteins are needed for the development of high-level resistance in zabofloxacin in *S. pneumoniae* [14]. In the present study, MIC results further confirmed that zabofloxacin gave the lowest quinolone MICs against ciprofloxacin-susceptible and ciprofloxacin-resistant *S. pneumoniae* strains (Table 1, Table 2 and Table 3). Zabofloxacin also had rapid bactericidal activity against PRSP and QRSP (Figure 1). More importantly, the results of in vivo studies demonstrated that zabofloxacin exhibited the most potent protective effects against systemic infection and respiratory tract infection caused by penicillin-resistant *S. pneumoniae* (Table 4, Figure 2). These results imply that zabofloxacin is useful for clearance of bacteria that grow in the lungs.

A previous pharmacokinetics study has shown that the *C*_max_ (maximum serum concentrations), AUC_0–48_, (the area under the plasma concentration versus time curve (AUC) from the time of dosing to 48 hours post-dosing), and AUC_0–∞_ (the AUC extrapolated to infinity) parameters of zabofloxacin hydrochloride were 1.89 ± 0.49 mg/L, 11.11 ± 2.00 kg·h/L, and 11.29 ± 2.01 kg·h/L, respectively. The half-life (*t*_1/2_) time of zabofloxacin was 8.2 ± 1.3 h [15]. Analyses for phase 2 clinical trial dose selection for zabofloxacin proposed that daily doses of 366 mg zabofloxacin provides a probability of *f*AUC24h/MIC ratio of 30 (free drug divided by the MIC, PK-PD target attainment) is essentially 1.0 for MIC values of 0.03 mg/L (zabofloxacin MIC_90_ against PSSP and PRSP).

While our results demonstrated that zabofloxacin has potent in vitro and in vivo activities against clinical isolated *S. pneumoniae*, there are some limitations in this study. First, the number of tested isolates is quite small. In addition, the strains were obtained between the years 2001 and 2010 years. Therefore, these epidemiological values in this study cannot represent current epidemiological trends.

## 4. Experimental Section

### 4.1. Antimicrobial Agents

Zabofloxacin was provided by Dong Wha Pharmaceutical Co. Ltd. (Anyang, Korea). Ciprofloxacin, sparfloxacin, moxifloxacin, linezolid, and synercid were purified from commercial tablets by recrystallization, and determined to be >99.9% pure by high-performance liquid chromatography analysis. Gemifloxacin was obtained from LG Chemical Ltd. (Daejeon, Korea). Oxacillin, penicillin G, vancomycin, and erythromycin were purchased from Sigma Aldrich (St. Louis, MO, USA).

### 4.2. Bacterial Strains

For in vitro susceptibility studies, 84 QSSP strains (ciprofloxacin MICs ≤4.0 mg/L) were obtained from hospitals in Seoul (Korea) between 2001 and 2010. From the collection, we selected 22 QRSP strains (ciprofloxacin MICs ≥8.0 mg/L) and tested them using the agar dilution MIC test. For the murine systemic infection model, colonies of PRSP 1065 were selected by screening clinical isolates.

### 4.3. Antimicrobial Susceptibility Test

The MICs were determined by the two-fold agar dilution method as described in the guidelines of the Clinical and Laboratory Standards Institute (CLSI) [16]. In brief, test strains were grown for 18 h in Todd-Hewitt broth (THB, Difco, Detroit, MI, USA) supplemented with 0.5% yeast extract (Difco) for 18 h at 37 °C, and then diluted with the same fresh medium to a density of 10^7^ colony-forming units (CFU) per milliliter. Cultures were inoculated in Muller-Hinton agar (MHA, Difco) plates supplemented with 5% defibrinated sheep blood (Komed, Sungnam, Korea) containing serial dilutions of the antimicrobial agents using a multi-pin inoculator to yield 10^4^ CFU/spot. Plates were incubated at 35 °C for 18 h, and were examined for growth. *S. pneumoniae* ATCC 6305 was used as a control strain. The MIC was considered the lowest concentration at which growth on agar plates is completely inhibited, disregarding a single colony or a faint haze caused by the inoculum.

### 4.4. Determination of Resistance Mechanism

To examine the efflux mechanism, quinolone-resistant strains were inoculated on agar plates in the presence or absence of 10 mg/L reserpine (Sigma), as described previously [17]. By definition, an efflux mechanism exists when there is at least a 4-fold lower MIC in the presence of reserpine [17].

QRDR sequences in *gyrA*, *gyrB*, *parC,* and *parE* were amplified by polymerase chain reaction (PCR) using primers described previously [18]. PCR products were purified using AccuPrep™ PCR Product Purification Kit (Bioneer Co. Ltd., Daejeon, Korea), and then sequenced using a system from Solgent Co. Ltd. (Daejeon, Korea).

### 4.5. Time-Kill Analysis

The time-kill studies were performed using the CLSI M26-A method [19]. In brief, *S. pneumoniae* 18 (QSSP) and *S. pneumoniae* 107282 (QRSP) strains were cultured in Muller-Hinton II broth (MHIIB, BD, Sparks, MD, USA) for 18 h at 37 °C. The cultured microbes were diluted with fresh MHIIB to a density of 10^5^ to 10^6^ CFU/mL and pre-incubated for 2 h. Then, zabofloxacin, ciprofloxacin, and gemifloxacin were added to the cultures at concentrations of 0.25×, 0.5×, 1×, 2×, and 4× MIC. The numbers of colony forming cells were quantified after 0, 2, 4, 6 and 24 h of incubation at 37 °C for 18 h by serial dilution on MHA. The compounds were considered bactericidal at the concentration that reduced the original inoculum by 3 log CFU/mL (99.9%) for each of the time periods.

### 4.6. Systemic Infection Model in Mice

Mice studies were performed as described previously [18]. *S. pneumoniae* strain was cultured in tryptic soy agar medium (Difco) supplemented with 5% defibrinated sheep blood at 35 °C for 18 h. For inoculation, *S. pneumoniae* 1065 was suspended in 0.9% NaCl. Groups of 6 male ICR mice (Dae Han Bio Link Co. Ltd., Eumseong-gun, Korea, weighing 18–22 g) were challenged intraperitoneally with 0.5 mL of the bacterial suspension, corresponding to an inoculum ranging from 10 to 100 times the minimal lethal dose (MLD) of the bacteria. Four dose levels were used for each antibiotic, depending on the in vitro antimicrobial activity of the compound. Zabofloxacin, ciprofloxacin, gemifloxacin, and moxifloxacin were administered orally to mice twice at 1 and 4 h post infection. Mice were housed in animal rooms maintained at 23 ± 2 °C with 55% ± 20% relative humidity. Mortality was recorded for 7 days, and the median effective dose needed to protect 50% of the mice (ED_50_) was calculated by the Probit method [20]. The challenge inoculum was sufficient to kill 100% of the untreated control mice, which died within 48 h post infection. Experimental protocols were approved by the Ethics Review Committee for Animal Experimentation at Handong Global University (Korea) (protocol #HGU-2008-01).

### 4.7. Respiratory Tract Infection Model in Mice

Penicillin-resistant *S. pneumoniae* 1065 strain was cultured in tryptic soy agar medium supplemented with 5% defibrinated sheep blood at 35 °C for 18 h. This strain was suspended in 0.9% NaCl. Male ICR mice (Dae Han Bio Link, weighing 18–22 g) were infected by intranasal route with 20 μL of *S. pneumoniae* 1065 suspension at a dose of approximately 10^7^ CFU/mouse. One day after the inoculation, the animals (in groups of four mice each) were treated with zabofloxacin or moxifloxacin orally once a day at a dose of 2 or 10 mg/kg for 3 consecutive days. The number of bacteria in the lungs was examined on the day following the final administration of the test drugs, namely, 4 days after inoculation. The lungs were removed aseptically and weighed, and then the viable bacterial counts were determined. Experimental protocols were approved by the Ethics Review Committee for Animal Experimentation at Handong Global University (Korea) (protocol #HGU-2008-01).

## 5. Conclusions

In this study, the results of in vitro and in vivo analyses strongly indicated that zabofloxacin is very effective in the treatment of pneumonia caused by multi-drug-resistant *S. pneumoniae*, including QRSP. Overall, these results imply that zabofloxacin is a promising fluoroquinolone with potent activity against clinically isolated *S. pneumoniae*.

## Figures and Tables

**Table 1:** Agar dilution minimum inhibitory concentrations (MICs) of 84 quinolone-susceptible *Streptococcus pneumoniae* strains (with ciprofloxacin MICs <4.0 mg/L).

Organisms (No. of Organisms)	Compounds	MIC_90_^a^	No. of Isolates with the Following MIC (mg/L)														
			0.008	0.015	0.03	0.06	0.125	0.25	0.5	1	2	4	8	16	32	64	>64
**PSSP ^b^ & PISP ^c^ (27)**																	
	Zabofloxacin	0.03	8	8	11												
	Ciprofloxacin	2							8	10	9						
	Sparfloxacin	0.5				3	7	8	9								
	Moxifloxacin	0.25			1	8	9	9									
	Gemifloxacin	0.03	8	5	13	1											
	Penicillin G	1	1	3	5		1	3	7	7							
	Oxacillin	8	2	1	1	4	1	2	3	5		1	5	2			
	Erythromycin	>64	3	3	6				2		2	2					9
	Linezolid	1						5	13	9							
	Synercid	1					1	11	2	13							
**PRSP ^d^ (57)**																	
	Zabofloxacin	0.03	8	30	19												
	Ciprofloxacin	2							4	29	24						
	Sparfloxacin	0.5				1	9	27	20								
	Moxifloxacin	0.25				5	29	23									
	Gemifloxacin	0.03	9	17	27	4											
	Penicillin G	4									27	30					
	Oxacillin	16									1	8	14	32	2		
	Erythromycin	>64			2	1		1	4	8	2		2			3	34
	Linezolid	1						10	17	30							
	Synercid	2						20	13	18	6						

^a^ MIC_90_: MIC at which 90% of the strains are inhibited; ^b^ PSSP: penicillin-susceptible *Streptococcus pneumoniae*; ^c^ PISP: penicillin-intermediate *S. pneumoniae*; ^d^ PRSP: penicillin-resistant *S. pneumoniae*.

**Table 2:** MICs of fluoroquinolones against 22 fluoroquinolone-resistant strains.

Quinolone	MIC Range	MIC_50_^a^	MIC_90_
Zabofloxacin	0.06–2	0.25	1
Ciprofloxacin	4–64	32	64
Moxifloxacin	2–8	4	8
Gemifloxacin	0.06–2	0.25	1

^a^ MIC_50_: MIC at which 50% of the strains are inhibited.

**Table 3:** MICs for 22 *Streptococcus pneumoniae* strains with defined mutations in the quinolone resistance-determining regions (QRDRs).

Strain No.	MIC (mg/L)				Detected Mutation(s) in QRDRs		
	ZAB ^a^	CIP ^b^	MOX ^c^	GEM ^d^	*gyrA*	*parC*	*parE*
104340	0.06	4	2	0.06	E85K	K136T	D435N
112519	0.125	4	2	0.06	S81F	S79F	- ^e^
104376	0.125	4	2	0.125	S81F	-	E474K
YS2	0.06	8	2	0.06	S81F	-	D435N, I460V
YS1	0.125	8	2	0.125	S81F	D83G, K137N	I460V
622286	0.25	8	2	0.125	S81F	D83N	-
102575	0.06	16	2	0.125	S81F	-	I460V
503167	0.125	16	4	0.06	S81F	S79F, K137N	I460V
103845	0.125	16	4	0.125	S81F	S79F	-
114794	0.125	16	4	0.25	S81F	S79F	I460V
102830	0.125	16	4	0.25	S81Y	D78N, S79F	-
102182	0.125	32	4	0.25	S81F	S79F, K137N	I460V
102239	0.25	32	4	0.5	S81F	S79F	I460V
102924	0.25	32	4	0.5	S81F	S79F	-
104710	0.25	32	4	0.5	S81F	S79F	-
107282	0.25	32	4	0.25	S81F	D83Y, K137N	I460V
113165	0.25	32	4	0.25	S81F	S79Y, K137N	I460V
SNU14	0.25	32	4	0.25	S81F	S79F, K137N	D435N, I460V
SNU17	0.25	32	4	0.25	S81F	S79F, K137N	P454S, I460V
120963	1	64 ^f^	8	1	E85K	S79F, K137N	-
103709	1	64 ^f^	8	1	S81F	S79F	-
103672	2	64 ^f^	8	2 ^f^	E85K	S79F	-

^a^ ZAB: zabofloxacin; ^b^ CIP: ciprofloxacin; ^c^ MOX: moxifloxacin; ^d^ GEM: Gemifloxacin; ^e^ -: mutations not detected; ^f^ Strains for which the MICs dropped at least four-fold in the presence of reserpine are indicated.

**Table 4:** Comparative in vivo activities of zabofloxacin against systemic infections in mice.

Microorganism (Inoculum)	Antimicrobial Agent ^a^	MIC (mg/L)	ED_50_ (mg/kg) ^b^ (95% Confidence Limits)
*Streptococcus pneumoniae* 1065 (PRSP) (2 × 10^7^ CFU/mouse) ^c^	Zabofloxacin	0.03	0.42 (0.04–1.62)
	Ciprofloxacin	4	31.45 (6.56–999)
	Moxifloxacin	0.25	18.00 (4.44–244)
	Gemifloxacin	0.03	2.0 (0.15–7.99)

^a^ Antimicrobial agents were orally administered twice at 1 and 4 h post infection; ^b^ ED_50_: median effective dose needed to protect 50% of the mice; ^c^ CFU: colony-forming units.
